# Impact of angina frequency on health utility values of patients with chronic stable angina

**DOI:** 10.1186/1477-7525-12-39

**Published:** 2014-03-14

**Authors:** Christine G Kohn, Matthew W Parker, Brendan L Limone, Craig I Coleman

**Affiliations:** 1 University of Connecticut School of Pharmacy, Storrs, CT, USA; 2 Department of Cardiology, Hartford Hospital, Hartford, CT, USA; 3 Evidence-Based Practice Center, University of Connecticut/Hartford Hospital, 80 Seymour Street, Hartford 06102-5037, CT, USA

**Keywords:** Chronic angina, Ranolazine, Seattle Angina Questionnaire, EQ-5D, Health utility

## Abstract

**Background:**

Chronic angina is a profoundly symptomatic disease. We evaluated the relationship between angina frequency and health utility.

**Methods:**

We used data from stable angina patients reporting ≥3 attacks/week enrolled in the Efficacy of Ranolazine in Chronic Angina (ERICA) trial. Angina frequency was classified using the Seattle Angina Questionnaire angina frequency (SAQAF) domain into no (100); monthly (61-99); weekly (31-60); and daily (0-30) angina. EuroQol (EQ)-5D health utility scores were derived from SAQ data using two mapping equations. Median EQ-5D utility scores for each SAQAF classification after the 6-week trial period were calculated (reported as: Equation 1/Equation 2). Changes in EQ-5D utility scores from baseline to end-of-trial for patients achieving and not achieving a ≥20-point improvement in SAQAF score and improving and not improving ≥1 SAQAF classification were compared.

**Results:**

Median EQ-5D utility scores (n = 548) were 0.68/0.60. Compared to patients reporting no angina symptoms (n = 28; 0.89/0.87) patients reporting monthly (n = 188; 0.80/0.76), weekly (n = 283; 0.72/0.65) and daily (n = 49; 0.65/0.54) symptoms had poorer health utility (p < 0.001 for both equations). Patients improving ≥1SAQAF classification (n = 254/541, 47%) experienced a median 0.05/0.07 greater improvement in EQ-5D health utility compared to those not improving ≥1 classification (p < 0.001 for both equations). Patients improving ≥20-points on the SAQAF (n = 355/541, 66%) experienced a median 0.06/0.07 greater improvement in health utility compared to those not achieving a ≥20-point improvement (p < 0.001 for both).

**Conclusions:**

Chronic angina patient health utility decreases as angina frequency increases. Patients reporting clinically important improvement in angina frequency experience a tangible improvement in health utility.

**Clinical trial registration:**

NCT00091429

## Background

It is estimated that nearly 8 million people in the United States (US) suffer from chronic stable angina [1]. Previous studies have demonstrated chronic stable angina has a significant negative impact on health-related quality-of-life (HrQoL) due to the bodily pain, psychological distress, impaired functioning, activity restriction and inability to care for oneself that is common to the disease [2].

The Seattle Angina Questionnaire (SAQ) [3] is a coronary disease-specific patient reported outcome measure (PROM) that has been included as a key endpoint in numerous clinical trials of stable coronary disease [4-9]. Some of these trials have published analyses evaluating morbidity, mortality and healthcare utilization across different frequencies of angina attacks; often categorizing patients as having no, monthly, weekly or daily angina symptoms based upon the angina frequency domain [8-11]. These analyses provide valuable insight into the relationship between angina frequency and event rates, healthcare utilization and treatment costs; however, in order to conduct a thorough cost-effectiveness analysis using these data, an assessment of patient health utility [values of preference for a disease state on a scale of 1.0 (perfect health) to 0.0 (death)] using matching angina frequency groupings are required [12].

There is currently a paucity of data describing relative health utility values for persons with chronic stable angina reporting the abovementioned frequencies of angina attacks. In these situations, guidance from the National Institute of Health and Care Excellence (NICE) [13] supports the use of mapping equations to translate responses from a PROM to an appropriate health utility tool (e.g. EuroQoL [EQ]-5D) [14]. In this study, we used two previously published mapping equations to convert individual patient SAQ data from a randomized controlled trial of chronic stable angina [4] into EQ-5D health utility scores [15,16] to evaluate the relationship between angina frequency and health utility.

## Methods

Data used in this post-hoc analysis were from the multi-national, double-blind, randomized, placebo-controlled, parallel group Efficacy of Ranolazine in Chronic Angina (ERICA) trial (clinicaltrials.gov registration: NCT00091429) [4]. The ERICA trial was approved by the institutional review board at each participating hospital, and each patient provided written informed consent. ERICA evaluated the effectiveness of ranolazine (1,000 mg twice daily) in adults with a documented history of coronary disease, at least a 3-month history of chronic stable angina, and reporting ≥3 episodes of angina/week during a 2-week qualification period despite treatment with 10 mg/day of amlodipine.

The SAQ was a secondary outcome measure administered at baseline and at the end of the 6-week double-blind full-dose trial phase. The SAQ is a validated, coronary disease-specific PROM comprising 19 items that quantify 5 clinically relevant domains of health status (angina frequency, physical limitation, angina stability, treatment satisfaction and disease perception) each scored on a scale of 0 to 100, with higher scores indicating better health status [3]. For this analysis, we used the SAQ angina frequency (SAQAF) domain scores to classify patients into discrete angina frequency categories; with a score of 100 = no; 61-99 = monthly; 31-60 = weekly; 0-30 = daily angina symptoms [9,10,17].

Two unique and independently derived mapping equations were used to translate (or cross-walk) individual SAQ domain scores for patients in the ERICA trial to EQ-5D-based health utility scores [15,16]. The first equation was derived in nearly 3,000 participants of the Alberta Provincial Project for Outcome Assessment in Coronary Heart Disease (APPROACH) database using linear regression within a Bayesian framework and scoring the EQ-5D using the US scoring algorithm (which ranges from −0.11 to 1.0 on a scale where 0.0 = death and 1.0 = perfect health). The equation [EQ-5D = 0.0010*(angina frequency domain score) – 0.0002*(angina stability domain score) + 0.0023 (disease perception domain score) + 0.0019 * (physical limitation domain score) + 0.0004 * (treatment satisfaction domain score) + 0.4388] uses all 5 domains of the SAQ to estimate EQ-5D health utility scores with a mean absolute error (MAE) of 0.088 (adjusted R^2^ = 0.37). The second equation used patient-level data from 5 studies of cardiac interventions, 3 of the 5 SAQ domains along with patient demographics, ordinary least squares linear regression methods and the United Kingdom (UK) scoring algorithm for the EQ-5D (which ranges from −0.594 to 1.0 on a scale where 0.0 = death and 1.0 = perfect health) to derive the following equation: EQ-5D = 0.002*(age) - 0.009 (if male) + 0.021(if medically managed) + 0.048 (if pre-percutaneous coronary intervention (PCI)) + 0.018 (if post-PCI) + 0.073(if pre-coronary artery bypass grafting) + 0.0036 *(physical limitation domain score) + 0.0021* (disease perception domain score) + 0.0015 * (angina frequency domain score) + 0.147 to estimate EQ-5D index scores with a MAE of 0.123 (adjusted R^2^ = 0.44).

Our analysis included all patients randomized in the ERICA trial that received at least one dose of double-blind investigational drug, had at least one subsequent primary efficacy assessment during the double-blind trial period and had a complete SAQ assessment at randomization and at the end-of-trial.

We estimated median (along with 25%, 75% ranges) EQ-5D health utility scores at randomization and at the end-of-trial for each patient using the above-mentioned mapping equations. Comparison of EQ-5D health utility scores across the four SAQ-based angina frequency classifications were made using end-of-trial values only (as no subjects were classified as having no angina symptoms and few were classified as having monthly symptoms at baseline due to trial inclusion criteria). We calculated change in EQ-5D health utility scores from randomization to end-of-trial and compared these values between subjects improving and not improving ≥1 SAQAF classification. Additionally we compared the change EQ-5D health utility scores for subjects achieving and not achieving a ≥20-point improvement in SAQAF domain score; a threshold previously reported as the minimally important clinical difference on the SAQAF domain score [11].

Categorical data were compared using chi-squared tests. Continuous data were compared using a Mann–Whitney U or Kruskal-Wallis one-way analysis of variance test, where appropriate. A p-value <0.05 was considered statistically significant in all situations. All analyses were conducted using SPSS version 20.0 (SPSS Inc., Chicago, IL, USA).

## Results

Characteristics of the 565 subjects randomized in the ERICA trial have been published previously [4]. We briefly summarize their key characteristics in Table 1.

**Table 1:** **Demographics, baseline characteristics and medical history of patients enrolled in the ERICA trial**[4]

**Characteristics**	**Placebo + Amlodipine (n = 283)**	**Ranolazine + Amlodipine (n = 281)**
Age (years), mean ± SD	61.3 ± 9.0	62.0 ± 8.7
Gender (M/W),%	73/27	72/28
Race,%		
White	99	98
Black	1	1
Asian	0	<1
Geographic region,%		
Eastern Europe	97	97
North America	3	3
Concomitant use of LANs,%	43	46
Weekly rate of angina attacks,trimmed mean ± SE	5.68 ± 0.26 (n = 281)	5.59 ± 0.21 (n = 277)
Weekly rate of NTG consumption, trimmed mean ± SE	5.02 ± 0.33 (n = 281)	4.43 ± 0.26 (n = 277)
SAQ score, mean ± SD		
Angina frequency	40.0 ± 14.9 (n = 281)	40.6 ± 13.2 (n = 277)
Physical limitation	48.9 ± 17.3 (n = 276)	49.2 ± 17.4 (n = 271)
Anginal stability	57.2 ± 17.7 (n = 281)	54.7 ± 18.0 (n = 277)
Disease perception	41.5 ± 17.8 (n = 281)	41.6 ± 17.2 (n = 277)
Treatment satisfaction	75.4 ± 14.0 (n = 281)	74.6 ± 14.3 (n = 277)
History of unstable angina,%	98 (35)	100 (36)
History of CHF,%	145 (51)	146 (52)
NYHA functional class I	38 (13)	32 (11)
NYHA functional class II	86 (30)	99 (35)
NYHA functional class III	21 (7)	15 (5)
NYHA functional class IV	0	0
Diabetes mellitus,%	54 (19)	52 (19)
Insulin-dependent	2 (1)	11 (4)
Previous myocardial infarction,%	233 (82)	218 (78)
Previous CABG,%	34 (12)	28 (10)
Previous PCI	25 (9)	34 (12)
Intermittent claudication,%	32 (11)	39 (14)
Hypertension,%	257 (91)	246 (88)

CABG = Coronary Artery Bypass Grafting; CHF = Congestive Heart Failure; ERICA = Efficacy of Ranolazine in Chronic Angina; LAN = long-acting nitrate; NTG = nitroglycerin; NYHA = New York Heart Association; PCI = Percutaneous Coronary Intervention; SAQ = Seattle Angina Questionnaire; SD = standard deviation; SE = standard error.

Both SAQAF and derived EQ-5D health utility scores were available in 548 of the patients (97% of all randomized). At baseline, the total trial population reported median SAQAF domain score of 40 (40, 50) with 6%, 71% and 23% reporting monthly, weekly and daily angina symptoms, respectively. Median EQ-5D health utility scores were 0.68 (0.64, 0.73) and 0.60 (0.55, 0.66) at baseline based on Equation 1 and 2.

Median EQ-5D health utility scores for each of the a priori determined SAQAF classifications at end-of-trial are reported in Table 2. A statistically significant association was seen between angina frequency classifications and EQ-5D health utility scores; with more frequent angina resulting in decrements in health utility (p < 0.001 for association for both Equations 1 and 2).

**Table 2:** EQ-5D health utility scores at end-of-trial stratified by Seattle Angina Questionnaire Angina Frequency Classification based upon mapping equations 1 and 2

		**Mapping Equation 1#**			**Mapping Equation 2†**		
**SAQAF domain score**	**N**	**Median EQ-5D score**	**25%, 75% range**	P-Value*	**Median EQ-5D score**	**25%, 75% range**	P-Value*
Overall	548	0.75	0.69, 0.80	NA	0.68	0.62, 0.77	NA
No (100)	28	0.89	0.84, 0.92	Referent	0.87	0.77, 0.91	Referent
Monthly (61–99)	188	0.80	0.75, 0.85	<0.001	0.76	0.70, 0.81	<0.001
Weekly (31–60)	283	0.72	0.68, 0.76	<0.001	0.65	0.61, 0.70	<0.001
Daily (0–30)	49	0.65	0.61, 0.69	<0.001	0.54	0.52, 0.61	<0.001

EQ-5D = EuroQol 5-Dimension; N = number of patients; SAQAF = Seattle Angina Questionnaire Angina frequency.

*p < 0.001 for the overall association using a Kruskal-Wallis one-way analysis of variance test; p-values for angina frequency domain score comparisons to “no” angina (referent) using Mann–Whitney *U* test.

#Mapping Equation 1 = Wijeysundera 2011 [15].

†Mapping Equation 2 = Goldsmith 2010 [16].

**Figure 1:**
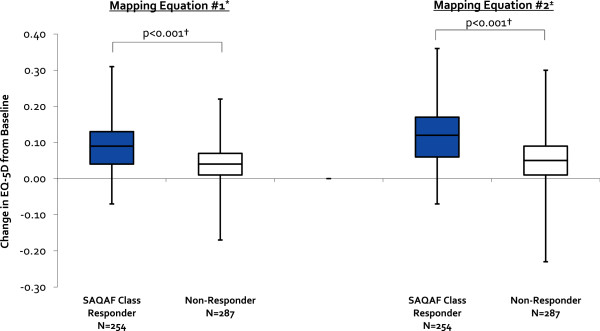
**Median Change in EQ-5D Health Utility Score From Baseline for Seattle Angina Questionnaire Angina Frequency Domain Score Class Responders and Non-Responders.** This figure presents box-and-whisker plots for the change in EQ-5D health utility score from baseline for SAQAF class responders (improving by at least one classification) and non-responders based upon data from both mapping equations. For each plot, the central box spans the first quartile to the third quartile (or the interquartile range), with the black line dividing the box depicting the median value for the subjects. The whiskers above and below the box show the minimum and maximum subject values. *Mapping Equation 1 = Wijeysundera 2011 [15]. ±Mapping Equation 2 = Goldsmith 2010 [16]. †p-values calculated using Mann–Whitney U tests.

**Figure 2:**
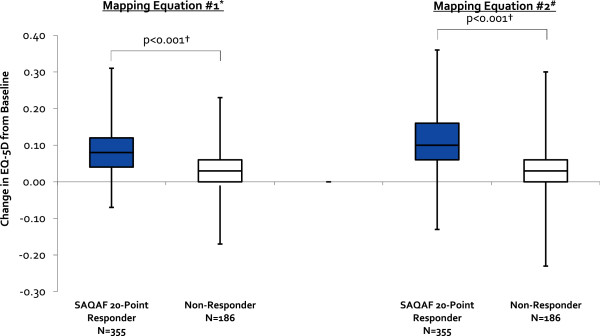
**Median Change in EQ-5D Health Utility Score From Baseline for Seattle Angina Questionnaire Angina Frequency Domain Score 20-Point Responders and Non-Responders.** This figure presents box-and-whisker plots for the change in EQ-5D health utility score from baseline for SAQAF 20-point responders and non-responders based upon data from both mapping equations. For each plot, the central box spans the first quartile to the third quartile (or the interquartile range), with the black line dividing the box depicting the median value for the subjects. The whiskers above and below the box show the minimum and maximum subject values. EQ-5D = EuroQol 5-Dimension; N = number of patients. *Mapping Equation 1 = Wijeysundera 2011 [15]. ±Mapping Equation 2 = Goldsmith 2010 [16]. †p-values calculated using Mann–Whitney U tests.

Patients who improved at least one SAQAF category from baseline (n = 254/541, 47%) experienced a median 0.05/0.07 greater improvement in EQ-5D health utility score compared to those not improving by at least one SAQAF classification (p < 0.001 for data based on both mapping equations) (Figure 1). Patients who improved ≥20-points on their SAQAF domain score from baseline (n = 355/541, 66%) experienced a median 0.06/0.07 greater improvement in EQ-5D health utility score compared to those not achieving a ≥20-point improvement (p < 0.001 for both equations) (Figure 2).

## Discussion

Using patient data from a randomized chronic stable angina trial [4] and 2 two different mapping equations [15,16], we have demonstrated that EQ-5D health utility values decrease significantly with worsening angina frequency categorization. The two independently derived mapping equations used in this analysis provided similar results; and depending on the equation used, patients regularly experiencing angina attacks reported clinically-relevant 0.07 (for monthly) to 0.33 (for daily) unit decrements (11% to 61% relative decrements) in health utility compared to patients reporting no angina. We also demonstrated patients improving by at least one SAQAF classification or reporting at least a 20-point improvement on the SAQAF domain score (previously estimated to signify a minimally important improvement) experienced a statistically significant and clinically-relevant improvement in health utility score. Thus, the above data suggests that appropriate management of stable angina symptoms can result in important improvement in patient HrQoL. In addition, our analysis provides the needed health utility values for stable angina patients with differing frequencies of angina symptoms required to calculate quality-adjusted life-years (QALYs) in cost-effectiveness (utility) analyses [12].

A previous analysis by the MERLIN –TIMI 36 investigators [16,18] (published in abstract form only) has also reported health utility values based upon the same SAQAF domain score categories we used. As in ours, this analysis demonstrated a strong and statistically significant (p < 0.001) association between angina frequency and health utility (“no” = 0.96; “monthly” = 0.81; “weekly” = 0.72; and “daily” = 0.65). Of note, unlike our own analysis, the MERLIN trial elicited health utility values by administering the EQ-5D tool to a large number of subjects (n = 5,388) 4-months after randomization. However, since MERLIN only included patients within 48-hours of a non-ST-segment elevation acute coronary syndrome, the reported health utility values may not fully represent those of a stable angina population [6]. Therefore, our analysis adds important information to current body of literature.

The two mapping equations [15,16] we used in our study to estimate health utility values had some important differences worthy of discussion. While both equations used SAQ domain scores to estimate EQ-5D health utility values; the equation by Wijeysundera and colleagues utilized all 5 SAQ domains, while the equation by Goldsmith and colleagues used only 3 (angina frequency, physical limitation and disease perception). Next, the equation by Goldsmith included demographic variables such as age, gender and use of PCI and CABG along with SAQ domains. By using this additional information, they were able to develop an equation that explained/predicted a greater proportion of the total variation in EQ-5D health utility scores evidenced by its higher adjusted R^2^ compared to Wijeysundera. However, a potential downside of including this data is that researchers wanting to utilize a mapping equation may not have access to some or all of these demographic variables. Finally, while the equation by Wijeysundera used the US algorithm to score the EQ-5D, Goldsmith used the UK scoring algorithm. Given this is a multi-national trial, neither equation is preferable; however, it is important to note that the scoring algorithms result in different potential ranges of values (−0.11 to 1.0 for the US and −0.594 to 1.0 for the UK equation) [14,15]. It is likely that each of the above-mentioned differences between the two mapping equations contributed to the differing EQ-5D health utility estimates arrived at by the two equations in our study.

There are some limitations to our analysis worth further discussion. First, since the SAQ domain scores required for mapping came from a single, moderately-sized randomized trial [4] that initially enrolled patients experiencing a relatively high frequency of angina and then treated these patients with an effective antianginal (ie, ranolazine), few patients finished the trial in the “no” or “daily” angina frequency categories. As a result, our estimates of health utility in these categories are associated with greater variance than the estimates in the ”monthly” and “weekly” groups with larger sample sizes. Next, as highlighted in the NICE guidance document [13], mapping is “at best, a second-best solution” to the direct collection of EQ-5D health utility values. However, in order to conduct a thorough cost-effectiveness analysis of stable angina interventions, an assessment of patient health utility using matching angina frequency groupings are required [12]. Unfortunately, there is a paucity of health utility data for discrete angina frequency categories in patients with chronic stable angina, and the ERICA trial did not utilize the EQ-5D or similar tool to elicit them directly. Consequently, our data likely represents some of the best estimates currently available. Finally, to address the potential short-coming of using any one mapping equation, we used multiple equations in this analysis to estimate a range of potential values.

## Conclusion

Chronic angina patient health utility values decrease as angina frequency increases. Our health utility estimates for stable angina patients stratified by angina frequency may be useful for conducting cost-effectiveness analyses in the future. Patients reporting at least a clinically important improvement in angina frequency experience a tangible improvement in health utility. Due to the strong relationship between these two outcome measures, future studies of chronic stable angina interventions should include health utility measures to aid in health economic evaluation.

### Consent

Written informed consent was obtained from all patients in this publication.

## Competing interests

Dr. Coleman has received honoraria for participation on advisory boards and research funding for Gilead Sciences. Drs Kohn, Parker and Limone have no conflicts to disclose.

## Authors’ contributions

Study concept and design: CGK, MWP, BLL, CIC. Acquisition of data: CIC. Analysis and interpretation of data: CGK, MWP BLL, CIC. Drafting of the manuscript: CGK, BLL, CIC. Critical revision of the manuscript for important intellectual content: CGK, MWP, BLL, CIC. Administrative, technical, or material support: CGK, CIC. Study supervision: CGK, CIC had full access to all the data in the study and take responsibility for the integrity of the data and the accuracy of the data analysis. All authors read and approved the final manuscript.
